# Establish a Pregnant Sow–Neonate Model to Assess Maternal Immunity of a Candidate Influenza Vaccine

**DOI:** 10.3390/vaccines11030646

**Published:** 2023-03-14

**Authors:** Fangfeng Yuan, Teresa Schieber, Tara L. Stein, Rachel M. Sestak, Callie J. Olson, Chi Chen, Victor C. Huber, Kelly Lechtenberg, Jodi McGill, Ying Fang

**Affiliations:** 1 Department of Pathobiology, University of Illinois Urbana-Champaign, Urbana, IL 61802, USA; 2 Department of Diagnostic Medicine and Pathobiology, Kansas State University, Manhattan, KS 66506, USA; 3 Midwest Veterinary Services, Inc., Oakland, NE 68045, USA; 4 Division of Basic Biomedical Sciences, Sanford School of Medicine, The University of South Dakota, Vermillion, SD 57069, USA; 5 Department of Veterinary Microbiology and Preventive Medicine, Iowa State University, Ames, IA 50011, USA

**Keywords:** influenza, vaccine efficacy, pregnant sow, maternal immunity

## Abstract

While it is well appreciated that maternal immunity can provide neonatal protection, the contribution of maternal vaccination toward generating such immunity is not well characterized. In our previous work, we created a candidate influenza vaccine using our chimeric hemagglutinin (HA) construct, HA-129. The HA-129 was expressed as part of a whole-virus vaccine that was built on the A/swine/Texas/4199-2/98-H3N2 backbone to generate the recombinant virus TX98-129. The TX98-129 candidate vaccine has the ability to induce broadly protective immune responses against genetically diversified influenza viruses in both mice and nursery pigs. In the current study, we established a pregnant sow–neonate model to evaluate the maternal immunity induced by this candidate vaccine to protect pregnant sows and their neonatal piglets against influenza virus infection. In pregnant sows, the results consistently show that TX98-129 induced a robust immune response against the TX98-129 virus and the parental viruses that were used to construct HA-129. After challenge with a field strain of influenza A virus, a significant increase in antibody titers was observed in vaccinated sows at both 5 and 22 days post challenge (dpc). The challenge virus was detected at a low level in the nasal swab of only one vaccinated sow at 5 dpc. Evaluation of cytokine responses in blood and lung tissue showed that levels of IFN-α and IL-1β were increased in the lung of vaccinated sows at 5 dpc, when compared to unvaccinated pigs. Further analysis of the T-cell subpopulation in PBMCs showed a higher ratio of IFN-γ-secreting CD4^+^CD8^+^ and CD8^+^ cytotoxic T cells in vaccinated sows at 22 dpc after stimulation with either challenge virus or vaccine virus. Finally, we used a neonatal challenge model to demonstrate that vaccine-induced maternal immunity can be passively transferred to newborn piglets. This was observed in the form of both increased antibody titers and deceased viral loads in neonates born from immunized sows. In summary, this study provides a swine model system to evaluate the impact of vaccination on maternal immunity and fetal/neonatal development.

## 1. Introduction

Influenza A viruses (IAV) pose a significant pandemic threat due to their ability to naturally infect both avian and mammalian hosts, including humans and pigs. Immunization remains the most effective method for influenza disease prevention, with the availability of live attenuated influenza virus (LAIV), inactivated influenza virus (IIV), and recombinant hemagglutinin (HA) vaccines [1]. During pregnancy, women have impaired immunity due to the formation of an immune-tolerant environment that prevents rejection of fetal tissues [2]. As observed during the 2009 H1N1 influenza pandemic, pregnant women are uniquely susceptible to severe influenza virus infections, which has increased attention to maternal immunizations [3]. Previous reports revealed that pregnant women are at an increased risk for influenza-associated complications and that infants born to severely infected women are at high risk for preterm birth and other adverse outcomes [4]. Despite the fact that pregnant women make up only 1% of the population, they accounted for 5% of deaths during the 2009 pandemic [5]. Due to this severe disease outcome observed during the 2009 H1N1 pandemic, the World Health Organization (WHO) set pregnant women as the highest priority to be vaccinated regardless of gestational age. Clinical trials in human populations showed that vaccination against influenza viruses reduces the risk of complications for pregnant women and adverse outcomes for infants [6]. Additionally, to date, there has been no evidence for maternal or infant complications related to the IIV vaccine [7].

Despite the fact that influenza vaccines are readily available each year and are recommended by the WHO, vaccine coverage has been historically lower than expected in the vulnerable populations [8]. Currently, data on influenza vaccine safety and efficacy during pregnancy are inadequate, which raises concerns regarding the effects of vaccines or medications on the fetus and thus contributes to lower vaccination coverage in pregnant women [9]. Initial risk–benefit assessment of maternal vaccination for prevention of viral infection requires a comparable animal model that provides in-depth guidance on influenza virus pathogenesis and vaccine efficacies. Since pigs are natural hosts for influenza virus, and they are similar in size, anatomy, physiology, organ development, and disease progression to humans [10], in this study, we established a pregnant sow–neonate model to evaluate a candidate influenza vaccine during pregnancy.

In our previous studies [11], we generated a candidate influenza vaccine, TX98-129, which expresses a chimeric HA (HA-129) derived from HAs of four genetically distinct swine influenza A viruses (H1N1) that had a history of zoonotic transmission. The chimeric HA was obtained through molecular breeding (gene shuffling) technology from the four original parental HAs. Our previous work showed that this candidate vaccine could induce broadly protective immunity against genetically divergent HAs in a nursery pig model [11]. In the current study, we assessed the effect of TX98-129 vaccine-induced maternal immunity on protecting pregnant sows and neonatal piglets against IAV infection. This study provides comparative information on the effect of the candidate influenza vaccine on maternal immunity. The pregnant sow–neonate model will be a highly useful comparative system for future evaluation of the effect of influenza vaccines, therapeutic drugs, and other treatments for the prevention of disease from infection during pregnancy, as well as studying their impact on fetal/neonatal development and subsequent consequences.

## 2. Materials and Methods

### 2.1. Cells and Viruses

Human embryonic kidney 293T cells [HEK-293T; American Type Culture Collection (ATCC), Manassas, VA] and Madin–Darby canine kidney cells (MDCK; ATCC) were maintained in minimum essential medium (Gibco, Waltham, MA, USA) supplemented with 10% fetal bovine serum (FBS), antibiotics (100 units/mL of penicillin and 100 mg/mL of streptomycin), and fungizone (0.25 mg/mL) at 37 °C with 5% CO_2_. The candidate influenza vaccine (TX98-129) contains a chimeric HA construct (HA-129) derived from HAs of four parental strains (TN09, NJ76, OH07, and IA06) using molecular breeding technology, while the other 7 gene segments were derived from TX98 virus (A/swine/Texas/4199-2/98) as described previously [11]. Phylogenetically, the four parental strains represent four distinct clades within H1 subtype, including TN09 from pdm clade, OH07 from γ clade, NJ76 from α clade, and IA06 from β clade. In addition, a field influenza virus isolate IL13 (A/swine/Illinois/A01158996/2013, H1N1) was obtained from National Veterinary Services Laboratories (NVSL) at Ames, IA, USA. The virus was originally isolated in a lung sample from a swine farm (sample received through the US National Swine Surveillance project). The passage 2 virus in MDCK cells was used for inoculating the pigs. The IL13 virus is closer to OH07 strain in γ clade, with 97.88% nucleotide identity with the HA gene, and shares 89.59%, 90.20%, and 89.72% identities with the HA of IA06, TN09, and NJ76 strains, respectively (GenBank accession numbers: KF313351-KF313358). The vaccine virus (TX98-129) and 4 reassortant parental viruses (TX98-TN09, TX98-NJ76, TX98-OH07, and TX98-IA06) were rescued using the 8-plasmid reverse genetics system by incorporating co-cultured MDCK and HEK-293T cells [12]. The vaccine virus (TX98-129) was prepared as a formalin-inactivated vaccine as described previously [13]. The rescued four reassortant parental viruses and the field isolate IL13 were propagated in MDCK cells in the presence of TPCK-trypsin (Thermo Fisher Scientific, Carlsbad, CA, USA) as described previously [14]. 

### 2.2. Pig Experiment

To assess vaccine efficacy in pregnant sows, we obtained 16 pregnant sows that were free of specific pathogens, including swine influenza virus and porcine reproductive and respiratory syndrome virus. Sows with no prior pregnancies were selected and artificially inseminated. They were randomly divided into four groups, and each group was housed in an individual isolation room in Animal Biosafety Level 2 (ABSL-2) facility. Groups B and C were vaccinated intramuscularly with 200 µg/mL of inactivated TX98-129 at 60 and 75 days gestation (DG), while groups A and D were mock-vaccinated with PBS as control (Table 1). All sows were challenged with either PBS or 2 × 10^7^ TCID_50_ of virulent swine influenza A virus (IL13; A/swine/Illinois/A01158996/2013) at 90 DG. Fetuses were collected at two time points, 5 days post-challenge (DPC; 95 DG) and 22 DPC (112 DG), before farrowing. Clinical signs were monitored daily. Blood and nasal swab samples were collected at 0 days post vaccination (DPV), 0 DPC (90 DG), 5 DPC (95 DG), and 22 DPC (112 DG). At necropsy, bronchoalveolar lavage fluid (BALF) samples were collected. Gross lung lesions were evaluated by an experienced pathologist using a method described previously [15]. The body weight of each fetus was recorded during necropsy, and average body weight of fetuses in each group of sows was calculated by combining the body weight of fetuses from both 5 DPC and 22 DPC and then dividing by the number of total fetuses in each sow group.

To evaluate vaccine-induced passive immunity against parental strains, 8 pregnant sows (Group 1–8) at 65 DG were obtained and housed in separate rooms in the ABSL2 animal isolation facility at the University of Illinois (Table 2). In total, 4 sows were vaccinated with the inactivated TX98-129 vaccine at 70 and 90 DG, while the remaining 4 sows were mock-vaccinated with PBS (Table 2). At 6 days post farrowing (DPF), each group of newborn piglets was challenged intranasally with 2 × 10^7^ TCID_50_ of individual parental viruses (TX98-IA06, TX98-NJ76, TX98-OH07, and TX98-TN09). Sows and neonatal piglets were euthanized at 5 DPC. Clinical signs and body temperature of neonatal pigs were observed daily. Blood samples from the sows were collected at 70 and 90 DG. Blood and nasal swab samples from neonatal piglets were collected at 0, 2, 4, and 5 DPC. At necropsy, gross lung lesions were evaluated and BALF samples were collected from each fetus.

### 2.3. Quantitative RT-PCR

To evaluate viral load in nasal swabs and BALF samples, a USDA-validated IAV real-time PCR assay targeting viral Matrix gene was employed [16]. Briefly, viral genomic RNA was extracted (MagMAX-96 viral RNA isolation kit, Thermo Fisher Scientific, Carlsbad, CA, USA) and real-time PCR was performed (Path-ID™ Multiplex One-Step RT-PCR Kit, Thermo Fisher Scientific, Carlsbad, CA, USA) using an Applied Biosystems™ 7500 Real-Time PCR instrument. The cycling parameters were 48 °C for 10 min, 95 °C for 10 min, 45 cycles of 95 °C for 15 s, and 60 °C for 60 s. Serial 10-fold dilution of TX98-129 virus from 1 × 10^6^ to 1 × 10^1^ TCID_50_/mL was performed to establish a standard curve for quantitation of viral RNA load equivalent to TCID_50_/mL.

### 2.4. Hemagglutination Inhibition (HAI)

HAI assay of serum samples was performed according to a procedure described previously [17]. Briefly, serum samples were treated with Receptor-Destroying Enzyme (RDE) and serially diluted in 96-well round-bottom plates. After 4 HA units of individual vaccine or challenge viruses were added to diluted serum, samples were then incubated at 4 °C for 1 h, and 50 μL of 0.05% chicken red blood cells (CRBC) was added. Plates were incubated at room temperature for 30 min, and the hemagglutination inhibition titer was recorded as the reciprocal of the final serum dilution exhibiting hemagglutinin inhibition.

### 2.5. Cellular Cytokine Gene Detection

Peripheral blood mononuclear cells (PBMCs) were subjected to total cellular RNA isolation using an SV Total RNA isolation kit according to the manufacturer’s instructions (Promega, Madison, WI, USA). Contaminating cellular genomic DNA was removed by using a Turbo DNA-free kit (Invitrogen, Carlsbad, CA, USA). Specific cytokine genes were detected by using 1 ug total RNA for first-strand cDNA synthesis (Maxima First Strand cDNA Synthesis Kit, Thermo Fisher Scientific, Carlsbad, CA, USA), and real-time PCR was subsequently performed (TaqMan Fast Advanced master mix, Thermo Fisher Scientific, Carlsbad, CA, USA) to quantify the expression of mRNAs of IFN-α, IL-1β, IFN-γ, IL-15, and TNF-α (primer/probe sets from Applied Biosystems, Foster City, CA, USA). The relative quantification of gene expression was calculated by normalizing it to the level of the housekeeping gene GAPDH.

### 2.6. Flow Cytometry Analysis

T-cell subpopulations were analyzed as described previously [18]. Briefly, virus (1 MOI) was used to stimulate PBMCs prior to addition of GolgiPlug (BD Biosciences, San Jose, CA, USA) and brefeldin A (Sigma-Aldrich, St. Louis, MO, USA) during the last 12 h of stimulation. Subsequently, PBMCs were first surface-labeled with markers for CD3ε, CD4α, and CD8α, followed by treatment using Cytofix/Cytoperm™ Fixation/Permeabilization Solution Kit (BD Biosciences, East Rutherford, NJ, USA). Cells were further stained with IFN-γ or isotype control and analyzed using a FACSAria II flow cytometer (BD Biosciences, East Rutherford, NJ, USA) and FlowJo software (v10.8.1, Tree Star, Ashland, OR, USA). Percentages of specific lymphocytes in PBMCs were presented for each cell population.

### 2.7. Statistical Analysis

GraphPad Prism 6 (GraphPad, La Jolla, CA, USA) was used for statistical analyses, with one-way analysis of variance (ANOVA) followed by Tukey’s post hoc test. Mean values with standard deviation were presented, and statistical significance was indicated at *p* values of: *, *p* < 0.05; **, *p* < 0.01; ***, *p* < 0.001; ****, *p* < 0.0001. A general linear mixed-effects model was created by using the lme4 package in R (v4.2.1) to quantify the impact of vaccination and viral challenge on the body weight of fetuses.

## 3. Results

### 3.1. Effect of TX98-129 Vaccine on Protecting Pregnant Sows against Influenza Virus Infection

In our previous studies [11], we generated a candidate influenza vaccine (TX98-129), which expresses a chimeric HA antigen (HA-129) derived from HAs of four genetically distinct swine influenza A viruses (H1N1) that had a history of zoonotic transmission, namely TN09, NJ76, OH07, and IA06 [11]. In the current study, we assessed the efficacy of the inactivated TX98-129 vaccine in pregnant sows (Table 1). Initially, pregnant sows were vaccinated with TX98-129 and then challenged with a field H1N1 virus IL13. Two sows from each group were terminated at 5 DPC (95 DG) and 22 DPC (112 DG), respectively. 

**Figure 1:**
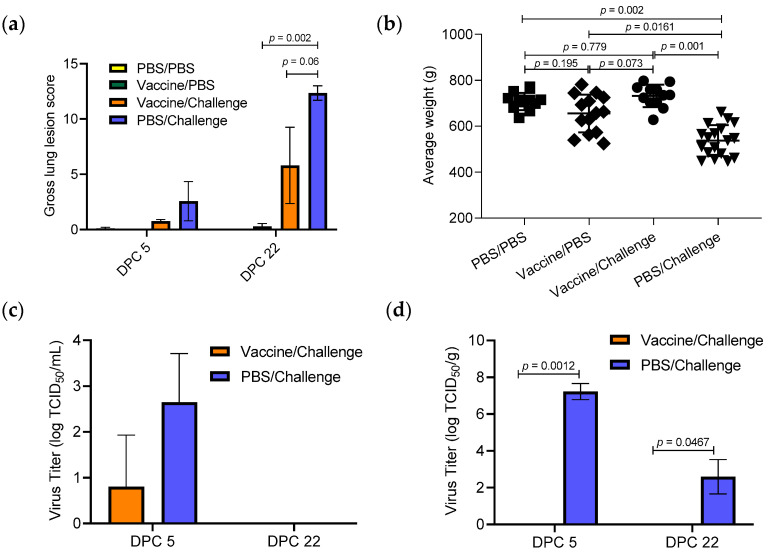
Clinical observations and viral titers after challenge. (**a**). Gross lung lesions of the sows at 5 DPC and 22 DPC. (**b**). Body weight of each fetus from different groups of sows. (**c**,**d**). Viral titers in nasal swabs (**c**) and lungs (**d**) from vaccine/challenge and PBS/challenge groups of sows at 5 DPC and 22 DPC. Statistical significance was determined by one-way ANOVA (Tukey’s test) and *p* value was indicated.

Sows were monitored daily, and no obvious clinical signs (coughing, sneezing, nasal discharge, fever) were observed after vaccination and after challenge with the influenza virus. At necropsy, lungs from the vaccine/challenge group of sows showed fewer gross lung lesions at DPC 5 (mean lesion score = 0.75) than the PBS/challenge group (mean lesion score = 2.58) (Figure 1a). Consistent with this result, at 22 DPC, the vaccine/challenge group had a mean lung lesion score of 5.8, which was lower than the mean lung lesion score (12.35) observed in the PBS/challenge group. The TX98-129 vaccine does not seem to cause lung lesions, as the vaccine/PBS group sows had no gross lesions observed at 5 DPC and a low lesion score of 0.28 at 22 DPC. The PBS/PBS control group sows were scored 0.1 at 5 DPC and 0 at 22 DPC. 

Fetus body weight from each sow was measured at necropsy. ANOVA analysis showed that the average body weight of fetuses was significantly lower in the PBS/challenge group of sows (548.23 g) when compared with those from the other three groups of sows (690.99 g for the PBS/PBS group, 661.56 g for the vaccine/PBS group, and 690.72 g for the vaccine/challenge group). In contrast, there was no statistical difference when comparing the average body weight of fetuses from the vaccine/challenge group of sows with those from the vaccine/PBS and PBS/PBS groups of sows (Figure 1b). Since the litter size varies between sows, we also analyzed the body weight by using a linear mixed-effects model. Again, there was no significant difference in the mean body weight between treatment groups of PBS/PBS, vaccine/PBS, and vaccine/challenge (Figure S1).

We further quantified the viral load in nasal swabs and BALF samples from the sows. Challenge virus was detected in nasal swabs from only one vaccinated sow at 5 DPC, with a titer of 10^1.6^ TCID_50_/mL (Figure 1c), while all pigs in the PBS/challenge group tested positive, with average viral titer of 10^2.5^ TCID_50_/mL at 5 DPC. No virus was detected in nasal swabs from either group at 22 DPC. When lung BALF samples were tested, challenge virus was detected in the PBS/challenge group at both 5 DPC (10^7.23^ TCID_50_/g) and 22 DPC (10^2.59^ TCID_50_/g), while the virus was not detected in the BALF samples collected from sows in the vaccine/challenge group (Figure 1d). Challenge virus was not detected in fetus samples collected from sows, including the vaccine/challenge and PBS/challenge groups of sows.

### 3.2. Protective Immunity Induced by TX98-129 Vaccine in Pregnant Sows

**Figure 2:**
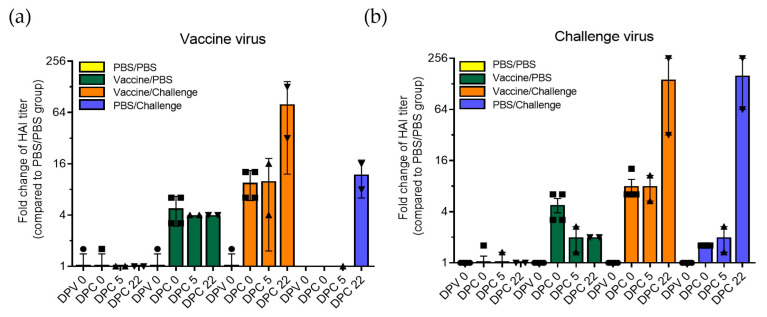
Comparison of HAI antibody titers in serum from different groups of sows. Four sows from each group were vaccinated twice with inactivated TX98-129 or PBS and further challenged with either influenza virus or PBS as control. Serum samples were collected at 0, 5, and 22 DPC and subjected to HAI assay against the vaccine virus (TX98-129, (**a**)) and challenge virus (IL13, (**b**)). HAI titers are defined as the reciprocal of the final serum dilution where inhibition of hemagglutination was observed. Fold-change of HAI titer compared to PBS/PBS group was calculated and plotted. Mean values and standard deviation of each time point of different treatment groups are indicated. Black circle, square, up-pointing triangle, and down-pointing triangle represent serum samples collected from DPV 0, DPC 0, DPC 5, and DPC 22, respectively.

Serum samples collected at each time point were subjected to HAI assay to detect antibodies against both vaccine virus (TX98-129) and challenge virus (IL13). Results show that this candidate vaccine induced an antibody response against the TX98-129 vaccine virus, with an average titer of 1:180 at 0 DPC (4.8-fold higher than that of the control group) (Figure 2a). After challenge with the field isolate IL13, a significant increase in antibody titers against the vaccine virus was observed in vaccinated sows at 5 DPC (average HAI titer 1:200; 10-fold higher compared to that of the control group) and 22 DPC (average HAI titer 1:1600; 80-fold higher compared to that of the control group). Similarly, antibody titers against the challenge virus were 1:60 in vaccine/challenge sows at 5 DPC, while at 22 DPC, the titers reached to an even higher level (average HAI titer 1:1440; 144-fold higher compared to that of the control group) (Figure 2b). After challenge with the IL13, the antibody titers in the PBS/challenge group achieved a HAI titer of 1:240 (12-fold higher) against the vaccine virus and 1:1600 (160-fold higher) against the challenge virus at 22 dpc (Figure 2). It is worth noting that antibodies against the vaccine virus are induced at higher levels after vaccination (Figure 2a, green bars and orange bars at 0 DPC and 5 DPC), while antibodies against the challenge virus are induced at higher levels after challenge (Figure 2b, blue bars at 22 DPC). The highest HAI titers against both viruses are achieved in the vaccine/challenge group at 22 DPC (orange bars).

**Figure 3:**
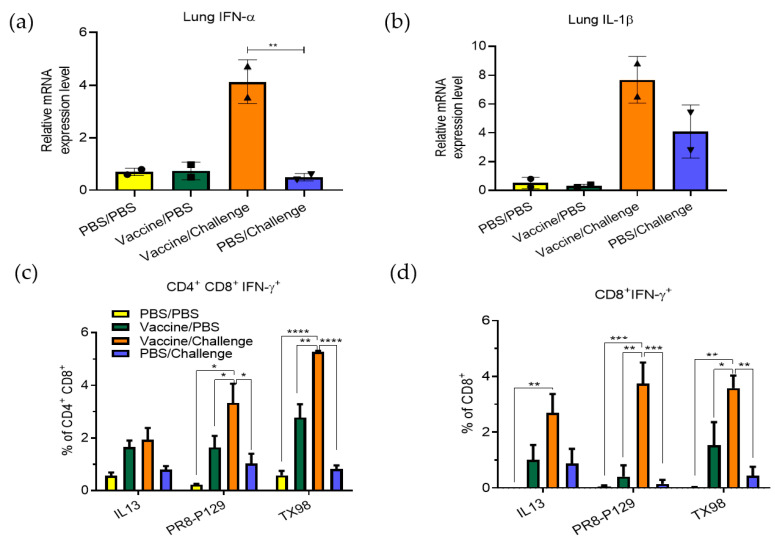
Innate and cellular immune responses after challenging with an influenza A virus field isolate. (**a**,**b**). Lung tissues collected from each group of sows at 5 DPC were subjected to cytokine RT-PCR analysis to measure the expression of IFN-α (**a**) and IL-1β (**b**) mRNA levels. Black circle, square, up-pointing triangle, and down-pointing triangle represent samples from treatment groups of PBS/PBS, vaccine/PBS, vaccine/challenge, and PBS/challenge, respectively. (**c**,**d**). PBMCs collected from each group at 22 DPC were stimulated with challenge virus (IL13), PR8-P129 recombinant virus, and TX98 backbone virus. Percent IFN-γ^+^ lymphocytes were calculated for CD4^+^CD8^+^ and CD8^+^ T-cells. Statistical significance was determined by one-way (**a**,**b**) or two-way (**c**,**d**) ANOVA (Tukey’s test) and is indicated with asterisks (*, *p* < 0.05; **, *p* < 0.01; ***, *p* < 0.001; ****, *p* < 0.0001).

Next, lung tissues collected from sows were analyzed for expression of IFN-α (Figure 3a) and IL-1β (Figure 3b). The results show that the levels of these cytokines were increased in the lungs of vaccinated sows at 5 DPC, when compared to those of unvaccinated sows. These cytokine responses may contribute to the observed viral clearance. We further analyzed virus-induced IFN-γ production by T-cell subpopulations using PBMC stimulated with three different viruses: (1) TX98 virus for immune responses against all viral proteins except HA-129; (2) PR8-P129 recombinant virus for responses against HA-129; and (3) the H1N1 IL13 isolate that was used as the challenge virus. The results show greater IFN-γ production by CD4^+^CD8^+^ (Figure 3c) and CD8⁺ cytotoxic T-cells (Figure 3d) in both the vaccine/PBS group and the vaccine/challenge group sows at 22 DPC after stimulation of PBMCs with different viruses. As expected, the PBS/challenge group and PBS/PBS group sows did not stimulate virus-specific T-cell responses at the levels observed in the vaccine groups.

### 3.3. Effect of Passive Maternal Immunity on Protecting Neonatal Piglets against Influenza Virus Infection

We further analyzed the protective immunity in newborn piglets obtained from passive maternal immunity of TX98-129 virus-immunized sows (Table 2). Eight pregnant sows were vaccinated with either PBS or inactivated TX98-129 vaccine. At six days post-farrowing, neonatal piglets were challenged with each of the four reassorted viruses (TX98-IA06, TX98-NJ76, TX98-OH07, TX98-TN09). All pigs were terminated at 5 DPC (11 DPF). 

**Figure 4:**
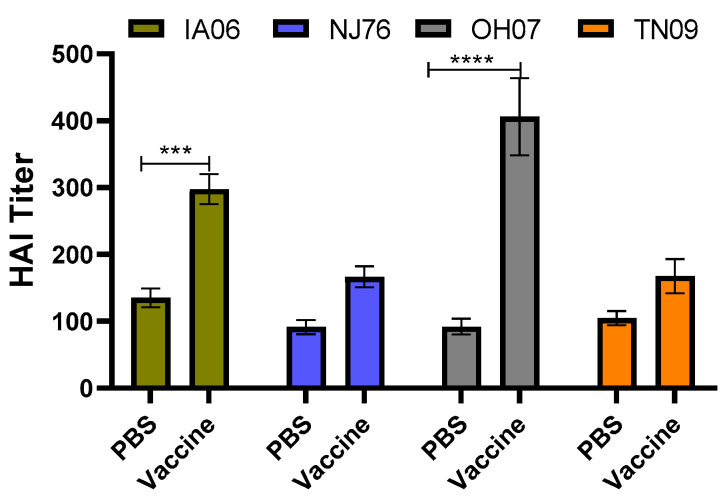
Serum antibody HAI titers from neonatal piglets infected with four parental viruses. Pregnant sows were vaccinated with either inactivated TX98-129 vaccine or PBS. Piglets born from the sows were challenged with a reassortant virus (TX98-IA06, TX98-NJ76, TX98-OH07, TX98-TN09). Terminal serum samples at 5 DPC were subjected to HAI assay against each of the challenge viruses. HAI titers are defined as the reciprocal of the final serum dilution where inhibition of hemagglutination was observed. Statistical significance was determined by one-way ANOVA (Tukey’s test) and is indicated with asterisks (***, *p* < 0.001; ****, *p* < 0.0001).

HAI assay was performed using serum samples from piglets at 5 DPC. When comparing groups of piglets challenged with the same virus, piglets of vaccinated sows showed higher serum antibody titers than those of unvaccinated sows (Figure 4). Specifically, there were significantly higher antibody levels against IA06 (*p* < 0.01) and OH07 (*p* < 0.001) virus in vaccine group piglets that were challenged with either TX98-IA06 or TX98-OH07 virus, respectively. In fact, the IA06 virus-challenged piglets from the vaccinated sows generated significantly higher antibody titers against all three challenge viruses, including TX98-IA06, TX98-OH07, and TX98-TN09, while the OH07 virus-challenged vaccine-group piglets produced significantly higher antibody responses against the TX98-OH07 and TX98-TN09 viruses (Figure S2). The TX98-NJ76 virus-challenged vaccine-group piglets only produced significantly higher antibody responses against the TX98-OH07 challenge virus. Interestingly, all of the virus-challenged vaccine-group piglets generated relatively lower-level antibody responses against the TX98-TN09 virus (Figure S2).

**Figure 5:**
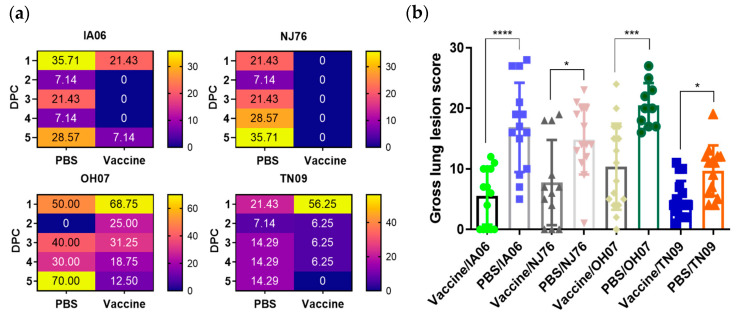
Clinical observations in neonatal piglets. (**a**) Percent of piglets displaying fever (>104 °F) after challenge with four reassortant parental viruses. Sows were inoculated with either vaccine or PBS, and neonatal piglets were challenged with each of the four reassortant parental viruses (TX98-IA06, TX-98-NJ76, TX98-OH07, and TX98-TN09). Rectal temperature was measured daily after challenge. (**b**) Gross lung lesions of piglets born from each individual sow. The X-axis shows each treatment group displayed by specific symbol with different colors. Statistical significance was determined by one-way ANOVA (Tukey’s test) and is indicated with asterisks (*, *p* < 0.05; ***, *p* < 0.001; ****, *p* < 0.0001).

With the relatively higher levels of HAI antibody response in the piglets from vaccinated sows, the clinical presentation of these piglets showed certain levels of protection from influenza virus infection. Piglets developed fever with a body temperature above 104 °F after challenge with each of the reassortant parental viruses (Figure 5a). Piglets from vaccinated sows appeared to be partially protected from fever after being challenged with TX98-NJ76 virus, compared to those piglets from unvaccinated sows, among whom an increased percentage (21.43% at 1 DPC to 35.71% at 5 DPC) developed fever. Similarly, fewer TX98-IA06-challenged piglets from vaccinated sows (7.14% at 5 DPC) developed fever compared to those from unvaccinated sows (28.57% at 5 DPC). Consistent with this result, a decreased number of piglets with fever (from 68.75% at 1 DPC to 12.5% at 5 DPC) were observed in the vaccine/OH07 group compared to the PBS/OH07 group (70% of piglets still had fever at 5 DPC). A similar pattern was also observed in the TX98-TN09 group (Figure 5a). At necropsy, lung evaluation showed that there were significantly lower gross lesion scores in piglets from vaccinated sows than those piglets from unvaccinated sows after challenge with each of the reassorted parental viruses (Figure 5b).

**Figure 6:**
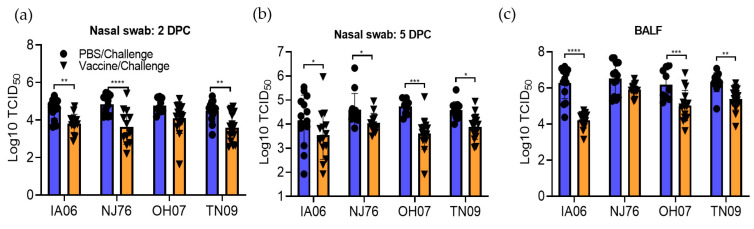
Viral load in piglets after challenge with influenza viruses. Influenza virus titers in nasal swab at 2 DPC (**a**), 5 DPC (**b**), and BALF (**c**) were determined by quantitative RT-PCR. Black circle and triangle represent PBS/challenge and vaccine/challenge groups, respectively. Statistical analysis for comparison between each challenge group was performed by one-way ANOVA. *, *p* < 0.05; **, *p* < 0.01; ***, *p* < 0.001, ****, *p* < 0.0001.

We further determined whether the higher levels of HAI antibody response in piglets correlated with the reduced viral load in nasal swabs and BALF samples. As we expected, quantitative RT-PCR results show that lower levels of viral shedding were observed in nasal swab samples of piglets from vaccinated sows compared to those from unvaccinated sows at 2 DPC and 5 DPC (Figure 6a,b). Consistent with this result, lower levels of viral load were also detected in BALF samples from piglets of vaccinated sows (Figure 6c).

## 4. Discussion

Preparedness for future influenza pandemics includes the consideration of the most vulnerable populations. Pregnant women are highly susceptible to complications from influenza virus infection due to impaired immune responses. Infections during pregnancy result in severe complications for both pregnant women and fetuses/infants. Although it is highly recommended that pregnant women are vaccinated promptly and take proper medications in the case of an influenza virus infection, a large proportion of individuals are still reluctant to comply with public health recommendations [9]. Influenza virus pathogenesis and vaccine efficacies in pregnant women have not been well studied due to limited pregnant animal models. In this study, we established a comparative pregnant sow–neonatal piglet model system to evaluate the effect of maternal immunity induced by a candidate influenza vaccine on protecting pregnant sows and their neonates from influenza virus infection. To our knowledge, the strategy of using pregnant sow/neonate as the animal model for influenza vaccine study is unprecedented. This comparative model system will be helpful to understand how influenza vaccines influence human maternal and fetal/neonatal immunity. The model also provides the means to study the impact of viral infection, drugs, therapies, and environmental factors on fetal/neonatal development and consequences.

The vaccine virus (TX98-129) used in this study was generated in our previous study, in which the chimeric HA (HA-129) was constructed by using molecular breeding technology with HAs of four phylogenetically distinct H1N1 parental viruses and was subsequently expressed by the backbone of TX98 H3N2 virus using reverse genetics [11]. This was designed in consideration of strain-specific immunity induced by seasonal influenza vaccines. There remains a need to develop novel influenza vaccines that induce broad immunity against heterologous field strains [19]. Previously, we demonstrated that administration of an inactivated TX98-129 candidate vaccine in nursery pigs was able to induce broad immunity against parental viruses and even other non-parental H1N1 subtypes [11]. In the current study, vaccination with inactivated TX98-129 vaccine in pregnant sows induced a robust antibody response against both vaccine and challenge viruses. The vaccine-induced passive immunity was able to (partially) protect neonatal piglets against infection by all four reassorted viruses that express the four distinct HA from parental viruses of HA-129, while HAI antibody levels vary in different piglet groups infected by different reassorted viruses, indicating specific as well as broad antibody responses. 

In addition to the specific antibody responses, we also observed increases in T-cell immune response (CD4^+^CD8^+^ and CD8^+^ cytotoxic T lymphocytes) in vaccinated sows. In addition to antibody-mediated immunity, CD8^+^ cytotoxic T lymphocytes are known to contribute to protective immunity against influenza virus infection [20]. Notably, influenza-virus-reactive CD4 and CD8 T-cells demonstrate broad antigen specificity and can recognize conserved internal proteins, such as matrix (M) and nucleoprotein (NP) [21]. Antibodies specific to NP and M1 proteins demonstrate effector functions against human influenza virus infections [22], and cross-reactive antibodies against NP that can mediate antibody-dependent cellular cytotoxicity have been included in strategies for developing universal vaccines [1,23]. The inactivated TX98-129 vaccine used in this study contains all viral components, of which the more conserved internal proteins could stimulate both antibody and T-cell responses to confer broad immunity. This correlates well with our result for the analysis of T-cell subpopulations in PBMCs. Both TX98 and PR8-P129 viruses stimulated higher frequencies of IFN-γ producing T-cells than that of IL13 field isolate. This result is expected, since the vaccine components contain the TX98 backbone and HA-129, and thus they provided identical or homologous stimulation. There are identical T-cell epitopes between the vaccines and the antigens used to stimulate T-cells in the antigen recall assays. In contrast, the IL13 virus is a field isolate that is heterologous to the vaccine antigens. While there are some shared T-cell epitopes, there are many internal T-cell epitopes present in TX98 and HA-129 are not present with the IL13 virus. On the other hand, because the IL13 virus is a field isolate, it is not as clearly characterized for *in vitro* assays. While we used 1 MOI of all viruses to stimulate the cells for the antigen-recall assays, the IL13 virus may behave differently (be more or less virulent) than the 1 MOI of TX98 or PR8-P129. Thus, its replication kinetics and capacity to stimulate the cells may vary. Regardless of stimulating viruses, our data clearly showed that the highest level of T-cell responses was observed in the vaccine/challenge group. 

Innate immune responses toward influenza virus infections play a key role by bridging early infection and onset of adaptive immunity [24]. Enhanced production and secretion of innate immune cytokines and chemokines are critical in limiting influenza virus infection [25,26,27]. In this study, we observed increased production of IFN-α in lung tissues of the sows after influenza virus challenge. It has been reported that all three types of IFNs expressed during the innate response of a virus infection are required for the development of robust adaptive immune responses to promote viral clearance [28]. In a previous study, pregnant women produced significantly less IFN-α and exhibited attenuated antiviral immunity against influenza virus infection, while vaccination significantly improved innate immunity during pregnancy [29]. Generally speaking, the inflammatory response triggered by influenza viruses is a double-edged sword [30]. Severe influenza virus infection leads to lung pathologies, which were believed to be the outcome of an aggressive proinflammatory response [31]. In our study, we observed that increased production of IFN-α and IL-1β in lung tissues was associated with reduced pathological lung lesions in vaccinated sows after virus challenge. Notably, the challenge virus is a field isolate, which appeared to have low virulence in infected sows with mild pathological lung lesions. Previous studies showed that the magnitude of innate immune activation varies based on the virulence of the challenge virus. Contemporary H1N1 influenza virus infections elicit transient activation of innate immune responses that ultimately contribute to virus clearance, while highly lethal viruses, such as the 1918 pandemic virus, trigger aberrantly high expression of innate immune proteins, including proinflammatory cytokines and chemokines, which causes tissue damages [32,33]. Future studies are warranted to test the effect of vaccines against IAVs at different degrees of virulence using the animal model system established in this study.

For the vaccination time point, we vaccinated sows at late gestation stage. This is in line with a previous clinical trial in pregnant women, which showed that the seroconversion rate was the highest (69.6%) when the vaccine was given during the late third trimester [34]. Fetal growth and organ development achieved a high level in the third trimester, and vaccination of pregnant mothers at this point could optimize neonatal antibody levels and ensure maximal vaccine safety for normal pregnancy [35]. However, another report showed that vaccination at any gestational age could yield an adequate antibody response to protect against influenza virus infection [36]. Further risk–benefit analysis of vaccination during all stages of pregnancy is warranted. This study established the model system for conducting more detailed time-course studies in the future to assess the safety and efficacy of vaccination throughout different stages of pregnancy.

Vertical transmission of influenza viruses has been controversial. In our study, virus was not detected in fetuses from any infected sows. This result is consistent with previous reports showing no evidence of placental transmission of H1N1 virus in either pregnant women [37,38] or mice [39]. However, another study reported evidence of cross-placental transmission by identifying influenza A virus (H1N1) in fetal tissues [40], while highly pathogenic strains of influenza virus, such as H5N1, are capable of transmitting across the placenta [37,41]. Future studies are needed to determine whether there is a stain/subtype-specific cross-placenta transmission of the influenza virus. 

## 5. Conclusions

In conclusion, we have established a pregnant sow–neonate model for influenza vaccine development. This model system will be an important tool to further study the impact of viral infection, drugs, therapies, and environmental factors on fetal/neonatal development in the future.

## Figures and Tables

**Table 1:** Design of pig experiment to assess vaccine efficacy in pregnant sows.

Group	Vaccine (60, 75 DG)	Challenge Virus (90 DG)	Fetus Collection (DG)
A (*n* = 4)	PBS	PBS	95 (*n* = 2) 112 (*n* = 2)
B (*n* = 4)	TX98-129	PBS	95 (*n* = 2) 112 (*n* = 2)
C (*n* = 4)	TX98-129	IL13	95 (*n* = 2) 112 (*n* = 2)
D (*n* = 4)	PBS	IL13	95 (*n* = 2) 112 (*n* = 2)

**Table 2:** Design of pig experiment to evaluate vaccine-induced passive immunity in neonatal piglets.

Group	Vaccine in Sows (70, 90 DG)	Number of Piglets after Farrowing (115 or 116 DG)	Challenge Virus in Neonatal Piglets (6 DPF)
1	PBS	14	TX98-IA06
2	PBS	14	TX98-NJ76
3	PBS	10	TX98-OH07
4	PBS	14	TX98-TN09
5	TX98-129	14	TX98-IA06
6	TX98-129	12	TX98-NJ76
7	TX98-129	16	TX98-OH07
8	TX98-129	16	TX98-TN09

## Data Availability

Data sharing is not applicable to this article.

## Supplementary Materials

The following supporting information can be downloaded at https://www.mdpi.com/article/10.3390/vaccines11030646/s1. Figure S1: Comparison of fetus body weight from different groups of sows.; Figure S2: Serum antibody HAI titers from neonatal piglets infected with four parental strains.
