# Supplementary material for: Establish a Pregnant Sow–Neonate Model to Assess Maternal Immunity of a Candidate Influenza Vaccine
Source: Vaccines (Basel). 2023 Mar 14;11(3):646. doi: 10.3390/vaccines11030646 (PMC10056052; doi:10.3390/vaccines11030646)
Supplement: Supplementary file 1 [file vaccines-11-00646-s001.zip › Vaccines-11-00646_Supplemental Figures.pdf]

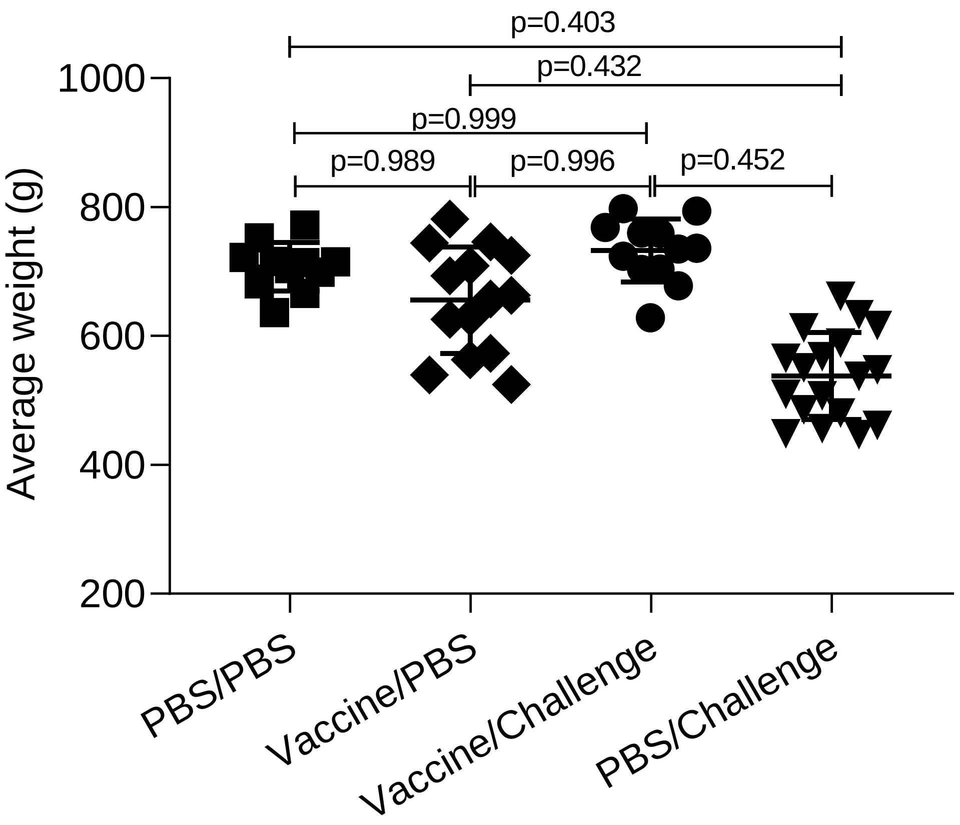

Figure S1. Comparison of fetus body weight from different groups of sows. A generalized linear mixed-effects model (GLMM) was used to analyze the impact of PBS, vaccination, and viral infection on the body weight of fetuses from each sow. P values are indicated in the graph. Calculations were made using R 4.2.1 and lme4 package.

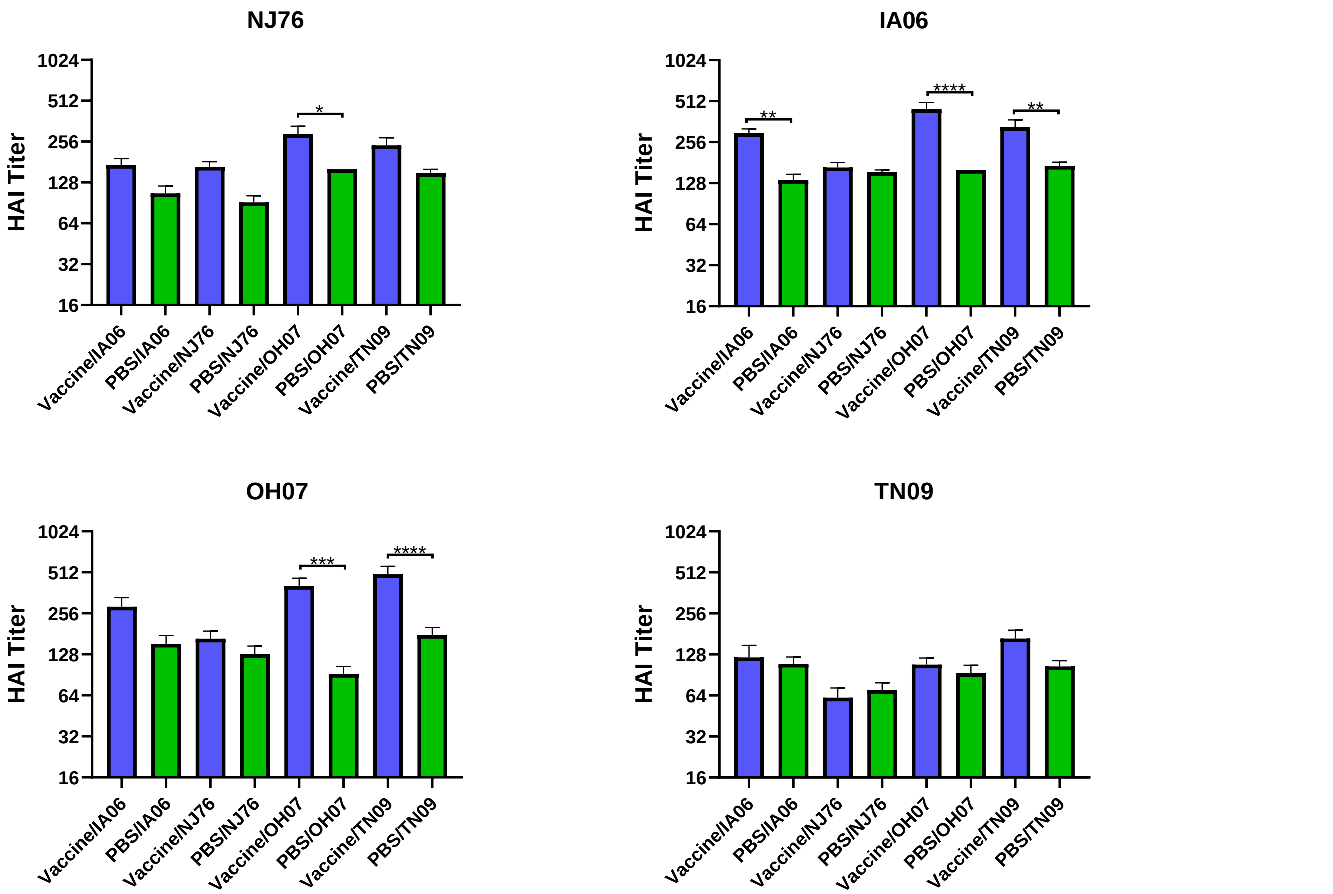

Figure S2. Serum antibody HAI titers from neonatal piglets infected with four reassortant parental viruses. Pregnant sows were vaccinated with either inactivated TX98-129 vaccine or PBS. Piglets born from the sows were challenged with four reassortant viruses (TX98-NJ76, TX98-IA06, TX98-OH07, and TX98-TN09). Terminal serum samples at 5 DPC were subjected to HAI assay against each of the challenge viruses. HAI titers are defined as the reciprocal of the final serum dilution where inhibition of hemagglutination was observed. Statistical significance was determined by one-way ANOVA (Tukey's test) and is indicated with asterisks (*, P<0.05; **, P<0.01; ***, P<0.001; ****, P<0.0001).
